# Carbonaceous Materials for Wastewater Treatment from Microwave-Assisted Pyrolysis of LignoForce Lignins

**DOI:** 10.1021/acsomega.5c11603

**Published:** 2026-06-08

**Authors:** Maria J. Suota, Mohammad S. Ghazani, Gorka Elordi, Jie Wu, Jack Saddler, Xiaotao Bi, Luiz P. Ramos

**Affiliations:** † Graduate Program in Chemistry, 28122 Federal University of Paraná, Curitiba, Paraná 81531-980, Brazil; ‡ Chemical and Biological Engineering Department, 8166 University of British Columbia, 2360 East Mall, Vancouver, British Columbia V6T 1Z3, Canada; § Chemical Engineering Department, 16402 University of the Basque Country (UPV/EHU), P.O. Box 644, Bilbao 48080, Spain; ∥ Department of Wood Science, Faculty of Forestry, The University of British Columbia, 2424 Main Mall, Vancouver, British Columbia V6T 1Z4, Canada

## Abstract

Microwave-assisted pyrolysis of kraft lignin was used to produce highly porous carbonaceous materials for environmental applications (biochars). LignoForce hardwood (LFHL) and softwood (LFSL) lignins were mixed with 30% potassium phosphate (K_3_PO_4_) and pyrolyzed at 450 °C to produce 45–47% biochar after 25 min of microwave irradiation. A 10 L min^–1^ nitrogen flow was used for purging during the pyrolysis and cooling. Elemental analysis (CHNS-O) revealed that kraft lignins were highly deoxygenated, but nearly 26% of their original oxygen content remained after pyrolysis, suggesting retention of oxygenated compounds in the biochar structure. Nitrogen adsorption (BET), scanning electron microscopy (SEM), and X-ray diffraction (XRD) demonstrated the formation amorphous micro- and mesoporous carbonaceous materials with a high potential for water and soil remediation. Significant specific surface areas (142 and 202 m^2^ g^–1^ for LFHL-B and LFSL-B, respectively) were obtained for these lignin biochars, which were tested for methylene blue (MB) adsorption at pH 4.5, 6.0, 7.5, and 9.0. LignoForce lignin biochars presented an adsorption capacity of approximately 15.5 mg g^–1^ and a removal efficiency higher than 94%, especially at higher pH levels (>4.5). However, MB adsorption was slightly faster for LFHL-B compared to LFSL-B. These results position kraft lignin as a valuable carbon-accumulating raw material for environmental applications and establish MAP as a fast and energy-efficient route for their thermal conversion.

## Graphical abstract

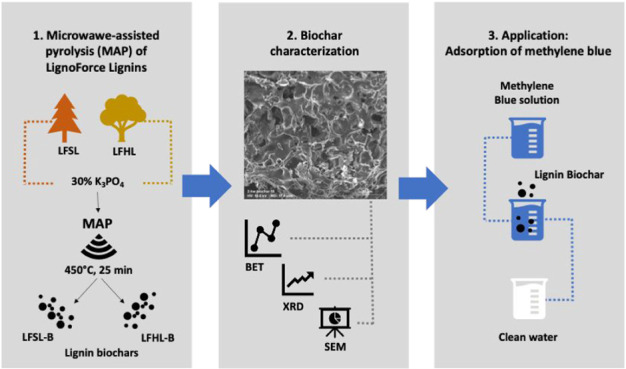

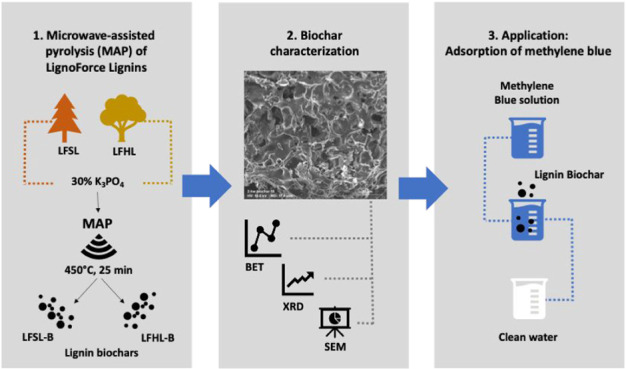

## 1 Introduction

A broad spectrum of sustainable products with great economic potential can be derived from lignin, such as biofuels, green solvents, adhesives, resins, biopolymers, biocomposites, platform chemicals, food and feed additives, and agricultural amenders.^1–3^ Beyond natural variation (e.g., hardwoods, softwoods, and herbaceous materials), industrial processes yield technical lignins (e.g., kraft lignin, sulfite pulping lignosulfonates, hydrolysis lignin, and organosolv lignin) with different properties (e.g., purity, solubility, and chemical functionality) that affect their reactivity toward developing advanced biobased materials.^2^ Among these, kraft lignin is a key renewable resource toward a sustainable bioeconomy because kraft pulping is the dominant process for producing wood pulp globally.^4,5^ Lignin has also been explored to produce highly adsorptive carbonaceous materials for environmental applications. This stems from its renewable nature, high carbon content, and the presence of phenolic structures that can be easily functionalized.^2,6,7^

Pyrolysis is a thermochemical process whereby organic precursors are heated in the absence of oxygen to produce a range of useful products—biochar, bio-oil, and gases—with distinct properties and applications.^8^ Biochar is a carbon-rich, porous material with modifiable surface functional groups that is ideal for environmental applications, including CCS (carbon capture and storage) and wastewater treatment, due to its high surface area and affinity for heavy metal ions and organic pollutants.^9–11^ Also, biochar holds significant potential in carbon farming. Its resistance to biological decay helps with long-term carbon storage in the soil, enhancing soil fertility and promoting sustainable agricultural practices.^12^ On the other hand, bio-oils contain a mixture of phenolic compounds and other organics that can be further processed into fuels or chemicals, while pyrolysis gases can be utilized for energy recovery or as feedstocks for chemical synthesis.^13^

Several types of pyrolysis have been described in the literature. These include conventional (slow, intermediate, fast, and flash) and advanced technologies such as catalytic, hydrothermal, plasma, and microwave-assisted pyrolysis (MAP).^14^ Compared to conventional processes, MAP offers advantages such as rapid and selective heating, faster reaction times, higher energy efficiency, better product homogeneity, and the ability to produce biochar with enhanced porosity and surface area.^10^ While conventional pyrolysis predominates in biochar synthesis from lignin for adsorption and catalytic applications, MAP has been oriented primarily toward bio-oil production.^15–17^ However, this technique has also been used to produce lignin biochars with enhanced porosity, large specific surface area, and improved performance for environmental applications.^18–20^

In general, MAP technologies require a microwave absorber to improve the heating rate up to pyrolysis temperatures. Silicon carbide, activated carbon, bentonite, clinoptilolite, potassium carbonate (K_2_CO_3_), and potassium phosphate (K_3_PO_4_) have been reported as suitable catalysts and microwave absorbers for MAP.^10,21,22^

K_3_PO_4_ seems to perform a dual role in the catalytic MAP. First, it functions as a highly efficient microwave absorber because of its high dielectric loss factor, producing ultrafast heating rates. Second, K_3_PO_4_ exerts a chemical catalytic effect by promoting lignin depolymerization, enhancing deoxygenation, and leading to higher yields of phenolic compounds compared to uncatalyzed pyrolysis.^23^ The presence of K^+^ and associated anions affects both primary and secondary reactions, thereby modifying the formation of various chemical species. For instance, potassium-based catalysts enhance the selectivity toward phenols and phenol derivatives in the catalytic pyrolysis of bean pods.^24^ Also, K_3_PO_4_, K_2_HPO_4_, or KH_2_PO_4_ inhibited the release of volatiles from holocellulose while promoting lignin conversion to phenolic compounds during the fast pyrolysis of poplar wood.^25^

The interaction of microwave energy with the lignin/catalyst mixture is critical for modifying lignin pyrolysis reaction pathways. The primary effect of microwaves is the promotion of high, localized, and selective heating rates (dielectric heating), providing a kinetic advantage compared to conventional heating. This directed energy significantly accelerates the kinetics of desirable primary cleavage reactions while suppressing undesirable secondary reactions, such as bio-oil repolymerization. These trends have been demonstrated for model compounds,^26^ where microwave energy was responsible for the selective cleavage of Cα–Cβ bonds in the phenylpropanoid side chains, releasing valuable aromatic monomers. This enhanced selectivity suggests that the ultrafast heating rates promote the preferential rupture of specific bonds, minimize secondary reaction pathways, and improve the selectivity toward aromatic monomers, resulting in a relatively simpler product mixture. Microwave energy has been identified as responsible for the selective cleavage of 96.3% of the Cα–Cβ bonds in organosolv lignin using methanol and ferric sulfate as a catalyst. The selectivity dropped to only 34% when the experiment was repeated under identical conditions using conventional heating.^26^

Biochar application for wastewater treatment has been studied extensively.^27–29^ Dyes are regarded as a threat to the ecosystem because of their recalcitrance and inappropriate disposal in natural watercourses. Even in low concentrations, dyes can be toxic, carcinogenic, and mutagenic, imposing several environmental risks when discharged into surface waters.^30^ Dye removal from polluted water is tricky because of its good solubility in water. Hence, the use of sustainable adsorbents has been a powerful alternative to remediate contaminated water from dye industries, including textiles. Methylene blue (MB) is one of the most used recalcitrant dyes in the textile industry.^31,32^ Within this context, lignin-derived biochars emerge as promising adsorbents for the sustainable, efficient, and cost-effective removal of dyes from watercourses.

The core novelty of this work is the unprecedented combination of MAP and potassium phosphate (K_3_PO_4_) with two commercial LignoForce lignins. Also, this is the first study to investigate and compare MAPs for both softwood and hardwood lignins. Our hypothesis was that, by comparing these two reference materials, the best lignin source will be identified to produce high yields of highly porous carbon materials with superior textural and improved adsorption properties for wastewater treatment. A comprehensive biochar characterization was performed and used to unlock the fundamentals of its high affinity for MB.

## 2 Materials and Methods

### 2.1 Materials

Softwood and hardwood LignoForce *kraft* lignin samples (LFSL and LFHL, respectively) were provided by FPInnovations (Canada). A full characterization of both LignoForce lignins has been reported elsewhere.^33^ Silicon carbide (SiC) and K_3_PO_4_ (98.7% purity) were purchased from Sigma-Aldrich (St. Louis, MO, USA), whereas MB (97% purity) was obtained from Fluka (Radnor, PA, USA). Other reagents, organic solvents, and standards were acquired in analytical grade and used as received.

### 2.2 Preparation of Pyrolysis Mixtures

The lignin powder (sieved to pass a 300-mesh screen) was mixed with commercial potassium phosphate (fine powder) and moistened with distilled water. After thorough homogenization, the mixture was sieved to form conglomerates with a size of approximately 10 mesh. The resulting granules were air-dried for 24 h until the complete elimination of the added water, which was monitored gravimetrically. Then, the final mixture, containing 30% potassium phosphate, was placed on a bed of silicon carbide (12 mesh).

### 2.3 Experimental Setup and Pyrolysis Experiments

The pyrolysis experiments were conducted in a microwave-assisted system manufactured by EnWave Corporation (Vancouver, Canada). The experimental setup consists of a 2.45 GHz magnetron to generate microwaves at 1000 W power, an *in situ* fixed-bed reactor with nitrogen (1.5 L min^–1^) purging at the bottom, and a three-stage condensation system to recover the pyrolysis liquids (Figure 1). This latter configuration featured two glass collection flasks kept in an ice box, linked to the base of three lateral condensers, with an additional acetone trap for further recovery. The gas phase was estimated by difference.

**Figure 1:**
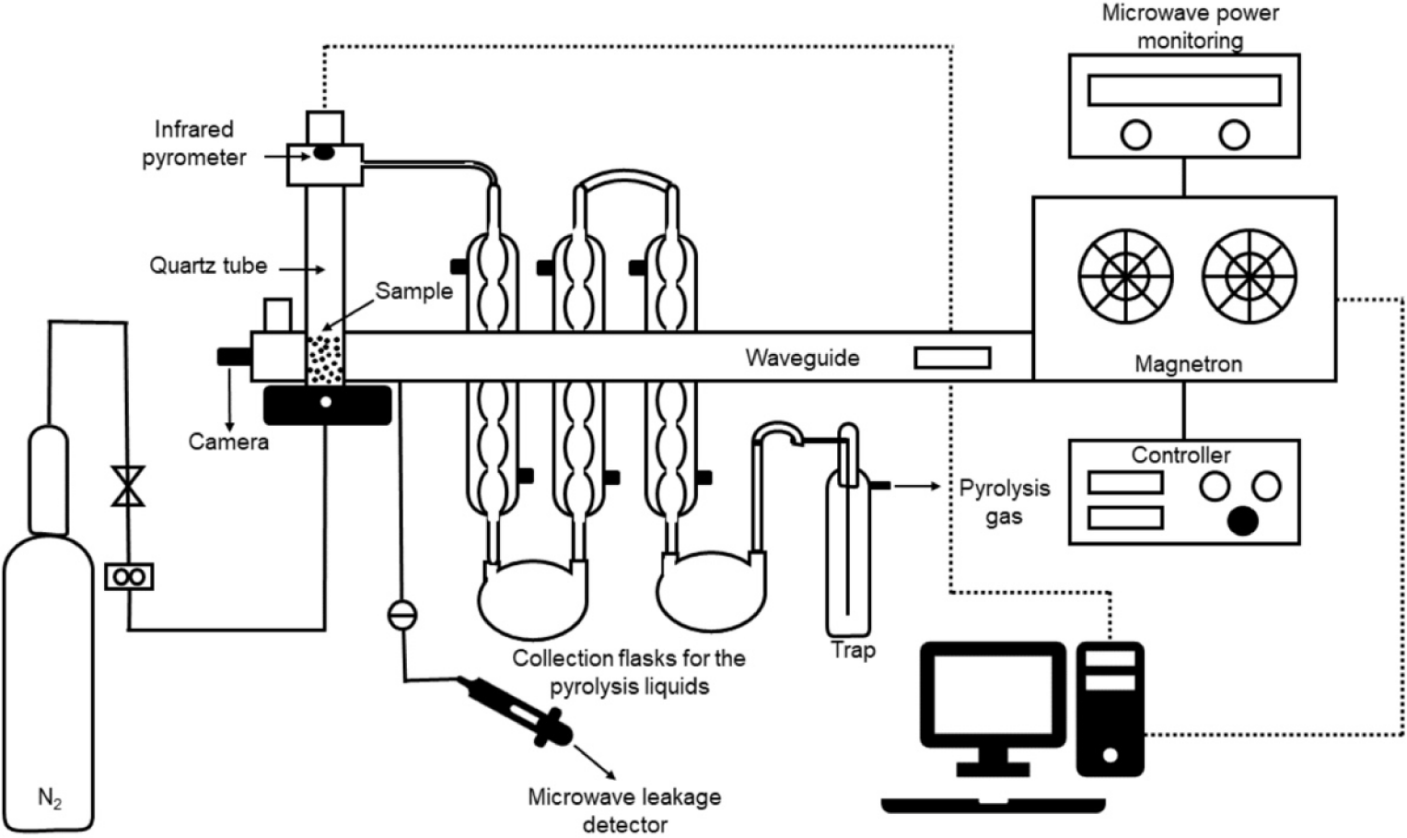
Schematic view of the microwave-assisted pyrolysis (MAP) unit.

The pyrolysis conditions (450 °C, 1000 W, and N_2_ flow of 10 Lmin^–1^) were selected based on previous studies of lignin-rich biomass, which identified this temperature as optimal for ensuring complete organic matter conversion while maximizing mass yield and good porosity. The experimental procedure is somewhat similar to what has been reported by Yang et al.^22^ and Zhong et al.^18^ In brief, lignin samples were premixed with 30 wt % K_3_PO_4_ and placed over a 20 g SiC bed inside a 44 mm quartz tube perpendicular to the microwave waveguide. An infrared pyrometer measured the temperature of the upper bed surface. The preloaded MAP system was purged with N_2_ at a 10 L min^–1^ flow rate. Then, the lignin-K_3_PO_4_ mixture (30 g) was irradiated with 100% of the microwave power (1000 W) until the temperature set point of 450 °C was reached. At this point, the microwave power was manually reduced to *circa* 80% to avoid overheating. Pyrolysis was performed for 25 min, after which the system was shut down and cooled to 24 °C under N_2_ flow. The resulting biochar was collected, weighed, and washed until the pH of the washing water reached 7. Bio-oil was recovered using acetone as the washing solvent for future investigation.

### 2.4 Elemental Analysis and Ash Content

Biochar elemental analysis was performed using an elemental analyzer from Elementar (Langenselbold, Germany), model Vario Micro Cube. C, H, N, and S contents were analytically determined in triplicate, and the O content was calculated by difference. The ash content was determined according to NREL/TP-510-42622.

### 2.5 Scanning Electron Microscopy (SEM)

The biochar morphology was examined using scanning electron microscopy (SEM) (S-3400N II, Hitachi, Japan). Samples were fixed in a self-adhesive sample holder to form a monolayer. Images were collected from different points after being exposed to an electron beam at an accelerating voltage of 10 kV.

### 2.6 Specific Surface Area and Pore Diameter Determinations

The biochar-specific surface area was measured from N_2_ adsorption/desorption isotherms at 77 K using a Micromeritics ASAP 2020 instrument (Georgia, USA). Samples (∼0.5 g) were vacuum-degassed at 300 °C for 16 h before analysis. Specific surface areas and pore diameters were determined by the Brunauer–Emmett–Teller (BET) method, whereas pore size distributions were based on the Barrett–Joyner–Halenda (BJH) method.

### 2.7 Methylene Blue Adsorption Tests

Methylene blue was used as a model compound for the adsorption experiments. First, the optimal biochar dosage was determined in 125 mL Erlenmeyer flasks using 0.025 to 0.450 g (equivalent to 0.5 to 9.0 g L^–1^) of biochar in 50 mL of a 50 mg L^–1^ methylene blue aqueous solution. The mixture was kept in an SI-300 Benchtop Shaker (Lab Companion, Daejeon, Republic of Korea) at 24 °C and 150 rpm for 2 h. Aliquots of 2 mL were withdrawn and centrifuged in an AccuSpin Micro17 (Fisher Scientific, Waltham, USA) for 10 min. The final concentration was measured by a UV–vis spectrophotometer (Cary 60, Agilent, USA) at 665 nm.

The biochar adsorption capacity (*q*, mg g^–1^) and removal efficiency (*E*, %) were calculated using eqs 1 and 2:

1 $$q=\frac{\left(Co-Ct\right)}{m}V$$

2 $$E=\frac{\left(Co-Ct\right)}{Ct}100$$

where *Co* and *Ct* are the initial and final MB concentrations (mg L^–1^), respectively, *m* is the adsorbent amount (g), and *V* is the total volume (L) inside the Erlenmeyer flask where the MB solution and the adsorbent were placed in contact.

The influence of pH on adsorption performance was determined in the pH range of 2 to 12 using 4 g L^–1^ biochar in 50 mL MB solution, following the same protocol as the dosing test. The pH was measured continuously using a pH meter, and the pH was controlled manually by adding NaOH or HCl solutions (1 mol L^–1^) dropwise to the MB solution.

### 2.8 Adsorption Isotherms

The MB adsorption isotherms were constructed through experiments that involved adding 0.150 g of biochar (LFHL-B or LFSL-B) to 50 mL of an MB solution with concentrations ranging from 5 to 100 mg L^–1^. MB adsorption was performed at 24 °C for 1 h under continuous magnetic stirring (150 rpm) and pH values of 4.5, 6.0, 7.5, and 9.0. The adsorption isotherms for LFHL-B and LFSL-B were fitted to the Langmuir, Freundlich, and Jovanović mathematical models to characterize the adsorption mechanisms and biochar surface properties. The Langmuir, Freundlich, and Jovanović exponential models are described in eqs 3, 4, and 5, respectively,

3 $$q_{e}=\frac{q_{m}K_{L}c_{e}}{1+K_{L}c_{e}}$$

4 $$q_{e}=K_{F}c_{e}^{1/n}$$

5 $$q_{e}=q_{m}\left[1-\exp\left(-K_{J}c_{e}\right)\right]$$

where *q_e_* (mg g^–1^) is the equilibrium adsorption capacity, *c_e_* (mg L^–1^) is the MB equilibrium concentration, *q_m_* (mg g^–1^) is the maximum adsorption capacity of the adsorbent, *K_L_* and *K_J_* (L mg^–1^) are the Langmuir and Jovanović constants, respectively, *K_F_* (mg g^–1^) is an adsorption capacity indicator, and *n* is an intensity indicator. The Langmuir, Freundlich, and Jovanović equations were applied to verify which models could genuinely represent the experimental results based on the sum of errors.

## 3 Results and Discussion

### 3.1 Heating Behavior for LignoForce Lignin Pyrolysis and Product Yield

Figure 2 shows the heating curves generated by MAP of LignoForce lignins. Microwave irradiation began at room temperature (∼24 °C) using 100% MW power (at 1000 W) and continued until 450 °C, when it was gradually reduced to 74% for LFHL and 79% for LFSL to stabilize the set point temperature. The plan was to reach the pyrolysis temperature and maintain a constant temperature by adjusting the microwave power. Both lignin samples exhibited similar behavior during the initial heating, taking approximately 34 to 36 s to reach 100 °C and remaining at that temperature for around 1.34 min. The small peak at this temperature corresponds to water removal, represented by step 1 in Figure 2.

**Figure 2:**
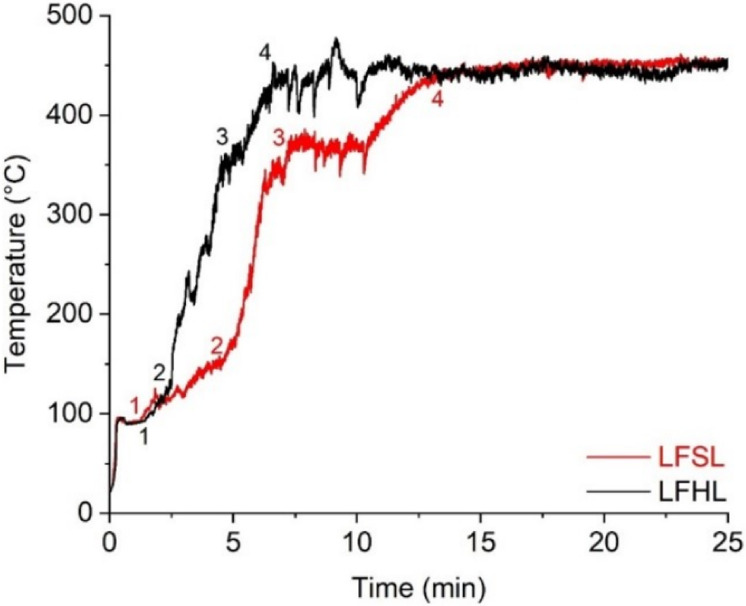
LFSL and LFHL heating curves for the catalytic microwave-assisted pyrolysis at 450 °C, 1000 W, N_2_ = 10 L min^–1^.

After drying, three additional heating stages were identified for both LignoForce lignins, shown by numbers 2, 3, and 4 (Figure 2). Each stage exhibits a different heating rate, which corresponded to 20.2 (1 to 2), 77.2 (2 to 3), and 11.5 °C min^–1^ (3 to 4) for LFSL and to 32.0 (1 to 2), 103.2 (2 to 3), and 45.7 °C min^–1^ (3 to 4) for LFHL. The overall heating rates were 31.2 and 54.0 °C min^–1^ for LFSL and LFHL, respectively, while the highest heating rates were determined by the corresponding slope of the curves between 2 and 3. A noticeable endothermic stage was identified for LFSL during the primary pyrolysis stage.

The thermal behavior of LignoForce hardwood and softwood lignins provided fundamental insights into the chemical and structural changes occurring during pyrolysis.^33^ TGA analysis of both LFSL and LFHL revealed a primary degradation stage between 250 and 480 °C, which was associated with the breakdown of labile chemical bonds, such as β-*O*-4’ aryl-ether linkages. This temperature range agrees with the target MAP temperature of 450 °C used in this study. TGA showed that this temperature is sufficient to trigger the highest mass losses by volatilization of low molar mass components. Also, LFSL stability was higher than that of LFHL, as evidenced by its higher onset (*T*_onset_ = 232.6 °C vs 212.8 °C) and glass transition temperature (*T*_g_ = 176.7 °C vs 131.7 °C), both consistent with their botanical origin.

Kawamoto^34^ highlighted that the roles of lignin linkages during pyrolysis are essential for understanding primary pyrolysis reactions. In general, α- and β-ether bonds were readily cleaved during the primary pyrolysis stage, whereas condensed (C–C’ type) linkages were stable during the thermal treatment of lignin model compounds. Likewise, LFSL and LFHL exhibited different heating profiles, which can be explained by the breakdown of different linkages in their compositions. LFHL has a β-*O*-4’ (C–O) content of 6.2 per 100 C_9_, while that of LFSL was 5.8. Furthermore, LFSL was found to have 68% more C–C linkages per 100 C_9_ (e.g., β–β’, β–1’, and β–5′) than LFHL.^33^ C–C bonds are more thermally stable than aryl-ether linkages, and their abundance in LFSL may explain the delayed temperature rise in its heating profile.

The MAP yield of solid, liquid, and gas products from lignin is shown in Figure 3. LFSL and LFHL both produced biochar as the primary product, with yields being slightly higher for the former compared to the latter. The higher concentration of guaiacyl (G) units in LFSL leads to a more condensed structure with stable C–C linkages (e.g., 5–5′), resulting in the higher biochar yields (47%) observed in our microwave experiments compared to LFHL (45%). Thermogravimetric analysis (TGA) showed that LFSL produced more biochar than LFHL under thermal conversion conditions.^33^

**Figure 3:**
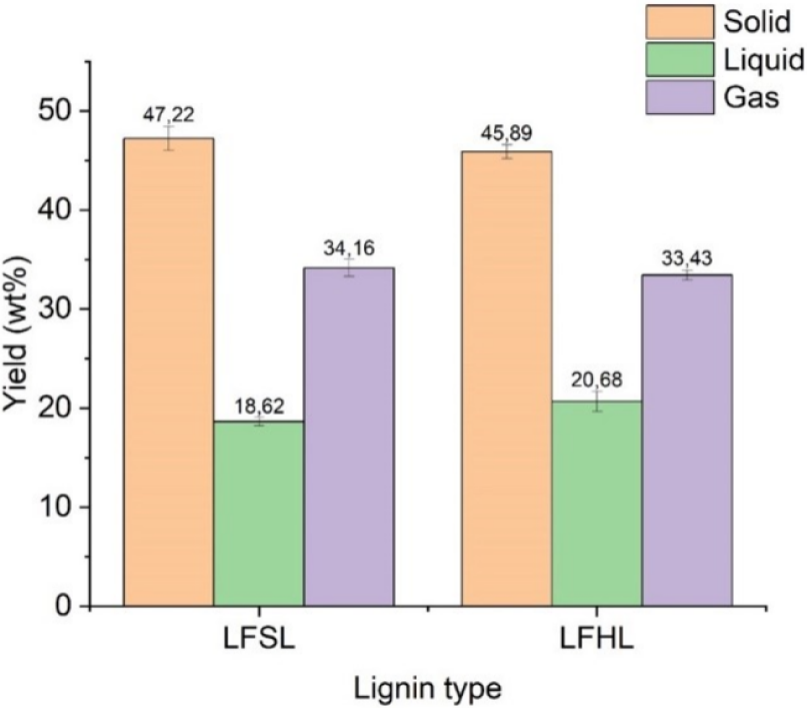
Mass yield of catalytic microwave-assisted pyrolysis products obtained from LignoForce lignins.

The yield of liquids for lignin samples was approximately 20 wt %, which is notably lower than what was reported by Fu et al.^16^ (36.1 wt %) during the MAP of softwood Lignoforce lignin. This can be attributed to the use of higher microwave power (1.5 to 2.7 kW) and the absorber type and ratio in their study (char, 40 wt %), which probably favored more intense heating compared to our study. Duan et al.^35^ reported yields of 22 wt % liquids and 58 wt % solids from commercial alkaline kraft lignin (from Sigma-Aldrich) using a 1000 W and 2.45 GHz microwave pyrolysis system, evidencing that the applied microwave power intensified the liquid product yield, likely due to its accelerated heating rate. Comparable data on hardwood lignin pyrolysis have not been found elsewhere.

Consistent with the literature, the yield of pyrolysis solids was higher than that of liquids and gases.^36^ However, higher temperatures and longer residence times inside the reaction chamber lead to lower biochar yields because the starting material is thermally decomposed into volatiles such as CO, CO_2_, CH_4_, and condensable molecules that make up the bio-oil composition.^37^ Therefore, optimizing the pyrolysis conditions and using an appropriate catalyst and reactor design can maximize biochar production. Mohamed et al.^23^ demonstrated that using pure K_3_PO_4_ (30 wt %) for switchgrass (*Panicum virgatum*) pyrolysis increased biochar yields compared to other pure (e.g., bentonite, clinoptilolite) or K_3_PO_4_-combined catalysts. Although yield increases may have been due to K_3_PO_4_ retention, K_3_PO_4_-rich biochars can enhance the water-holding capacity, ion exchange, and fertility of clayey sandy soils. Hence, the residual K_3_PO_4_ in the biochars may potentially improve soil properties in terms of micronutrient content, besides boosting crop productivity and reducing the need for chemical fertilizers.

### 3.2 Elemental Analysis and Ash Content

On the basis of our previous study, LFSL and LFHL had 64.50 and 62.85 wt % carbon in their composition, respectively.^33^ By contrast, their corresponding MAP biochars (LFSL-B and LFHL-B, respectively) had carbon contents of ∼87% (Table 1). Lower H/C and O/C elemental ratios in biochars indicate high carbonization compared with the lignin precursors. Low H/C and O/C ratios in Table 1 are also attributed to aromatization, which led to the formation of polyaromatic structures in biochar.^38^

**Table 1:** Elemental Composition of LignoForce Lignins and Biochars

	Composition (wt %)^a^					Atomic ratio		
Sample	C	H	N	S	O^b^	H/C	(O + N)/C	O/C
LFSL^c^	64.50	5.73	0.03	1.70	28.04	0.089	0.435	0.435
LFSL-B	87.83	1.93	n.d.^d^	n.d.	10.24	0.022	0.117	0.117
LFHL^c^	62.85	5.63	0.06	2.29	29.17	0.090	0.465	0.464
LFHL-B	87.67	1.57	n.d.	n.d.	10.76	0.018	0.123	0.123

a Based on ash and moisture-free values.

b Calculated by difference (O% = 100 – (C% + H% + N% + S%)).

c Based on our previous work.^33^

d n.d., not detected.

The reduction in the oxygen content in biochars suggests the degradation of oxygenated functional groups in lignin, leading to lower polarity and higher hydrophobicity. LFHL-B had an 18.2% lower H/C ratio than LFSL-B, indicating that it is more aromatic than the latter. Similar elemental compositions were observed for biochars produced from a mix of hardwood kraft lignin in a carbonization chamber (800 °C, 2 h, N_2_ at 100 mL min^–1^).^39^ The elemental composition of this biochar was reported as 84.4% C, 1.60% H, 0.99% N, and 8.67% O.

LFSL-B and LFHL-B had ash contents of 11.5 ± 0.3% and 11.4 ± 0.9%, respectively, consistent with the 12.8 ± 0.4% of alkaline lignin biochar that was produced at 700 °C in a tube furnace.^40^ LignoForce lignins, by contrast, contain approximately 1% alkaline ash.^33^ Therefore, both LFSL-B and LFHL-B retained K_3_PO_4_ in their composition which, by gradual leaching, could become a source of essential micronutrients for plant growth, boosting soil fertility and crop productivity.^23^ Hence, K_3_PO_4_-impregnated lignin biochars may perform as slow-release fertilizers, acting as soil conditioners and helping the recovery of degraded soils for precision agriculture.^41^

### 3.3 N_2_ Sorption Analysis

The N_2_ adsorption and desorption isotherms for LFSL-B and LFHL-B are shown in Figure S1 of Supporting Information. According to the IUPAC classification, the resulting profiles indicate a combination of types I and II isotherms. These isotherms suggest that biochar is a microporous material with pore diameters below 10 nm, including both wide and narrow mesopores.^42^ Zhu et al.^43^ identified micromesoporous structures in biochars derived from alkaline lignin via fast pyrolysis at 550 °C, followed by activation with KOH, employing the N_2_ sorption–desorption technique.

The BJH method was used to determine the pore size distribution of the LignoForce lignin biochars. Both LFSL-B and LFHL-B showed a well-developed pore structure (Figure S2). LFSL-B presented a better-structured porous surface, containing a specific surface area 30% greater than LFHL-B, with twice the external surface area (ESA) and total pore volume (TPV). Furthermore, both micropore area (MPA) and micropore volume (MPV) were 23% and 31% higher, respectively (Table 2). Therefore, a better adsorption performance is expected for LFSL-B due to its higher specific surface area. Usually, nonactivated lignin biochars produced between 400 and 500 °C have a specific surface area ranging from 3 to 30 m^2^ g^–1^,^44,45^ whereas the specific surface area can be higher than 300 m^2^ g^–1^ for activated lignin biochars or biochars produced at higher temperatures (*e.g.,* 600 °C).^44,46^ Zhong et al.^18^ produced high-quality biochars from softwood kraft lignin with a similar porous structure, although with a BET surface area 20% smaller than LFSL-B.

**Table 2:** Pore Structure for the LignoForce Lignin Biochars Determined by N_2_ Adsorption and Desorption at −77 K

Biochar	LFSL-B	LFHL-B
Specific surface area (S_BET)_ (m^2^/g)	202.6 ± 2.0	142.8 ± 0.5
Micropore area (MPA) (m^2^/g)	156.6	120.2
External surface area (ESA) (m^2^/g)	45.93	22.57
Total pore volume (TPV) (cm^3^/g)	0.14961	0.08257
Micropore volume (MPV) (cm^3^/g)	0.083	0.057
Average pore diameter (ADP) (nm)	3.19	2.75

The formation of a well-developed pore structure in biochars is highly dependent on the pyrolysis temperature, with higher temperatures generally resulting in better pore development regardless of the source. Li et al.^44^ demonstrated that lignin, cellulose, and pinewood biochars increased their specific surface area when the pyrolysis temperature changed from 400 to 600 °C. The present study employed a moderate pyrolysis temperature of 450 °C; thus, the resulting porosity in LFSL-B and LFHL-B must have been influenced by the combination of catalyst and microwave irradiation. Mixing the catalyst with lignin, followed by irradiation, favored the formation of hotspots throughout the material and homogeneous heating by microwave irradiation. Yang et al.^22^ explained that microwave radiation absorption by the sample (mainly the catalyst) generated heat, which was primarily transferred to surrounding sample particles by conduction, raising the temperature. The high MAP heating rates were favorable for the sudden elimination of volatile organic compounds and water, which is critical for creating well-developed porous structures.^47^ Jiang et al.^39^ obtained biochar with a specific surface area of 83.4 m^2^ g^–1^ by pyrolyzing hardwood lignin at a higher temperature (800 °C) and a heating rate of 10 °C min^–1^. Compared to conventional pyrolysis, MAP tends to produce biochars with a better pore structure due to its ability to achieve high heating rates.^22,47^ In addition, MAP biochars can be reused across multiple cycles because their micropore-rich structure ensures the structural stability necessary to withstand thermal activation and maintain long-term adsorption performance. According to Greiner, Shimabuku, and Summers,^20^ thermal regeneration promotes a correlated increase between BET surface area and adsorption capacity, which continues to grow even after a second exhaustion-regeneration cycle. However, while the specific capacity improves through this pore development, the overall adsorption efficiency does not increase due to the material mass loss incurred during the heat treatment.

### 3.4 Biochar Crystallinity

XRD was used to examine the presence of crystalline phases in LignoForce lignin biochars. LFSL-B and LFHL-B diffraction patterns (Figure 4) exhibit the predominance of a nanoscale amorphous material containing small peaks that can be associated with SiC particles.^48^ The upward flow of N_2_ probably entrained tiny SiC particles in both lignin biochars. Tam et al.^49^ reported broad peaks at 24.2°, 30.1°, and 31.3° of 2θ in the XRD of a softwood Klason lignin biochar, and nanoparticles were detected on its surface by SEM. Although crystalline graphite was not detectable, the 2θ positions of its diffraction peaks were highlighted as dashed lines, indicating the expected locations of its crystalline domains. Similarly, Zhu et al.^43^ produced alkaline lignin biochars using conventional pyrolysis at 550 °C and did not detect any graphitic domains by XRD, even after activation.

**Figure 4:**
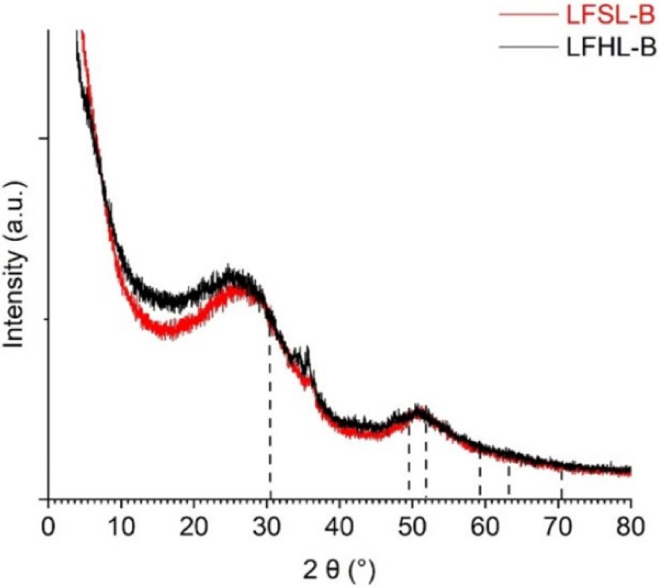
XRD patterns of LignoForce lignin biochars obtained by microwave-assisted pyrolysis (1000 W, 450 °C, 25 min, 30% K_3_PO_4_).

### 3.5 Biochar Morphology

The morphology of pyrolytic biochars results from the rapid evolution of vapors and gases during biomass thermal decomposition at high temperatures, creating pores with a wide range of sizes. In addition, surface irregularities and roughness can result from the evolution of volatiles.^47^ The uneven surfaces of the lignin biochars, containing spherical cavities of different sizes and depths, are shown in Figure 5. In general, LFSL-B and LFHL-B were morphologically similar. However, the LFHL-B presented lower mechanical resistance or higher brittleness, easily breaking when it was removed from the quartz tube.

**Figure 5:**
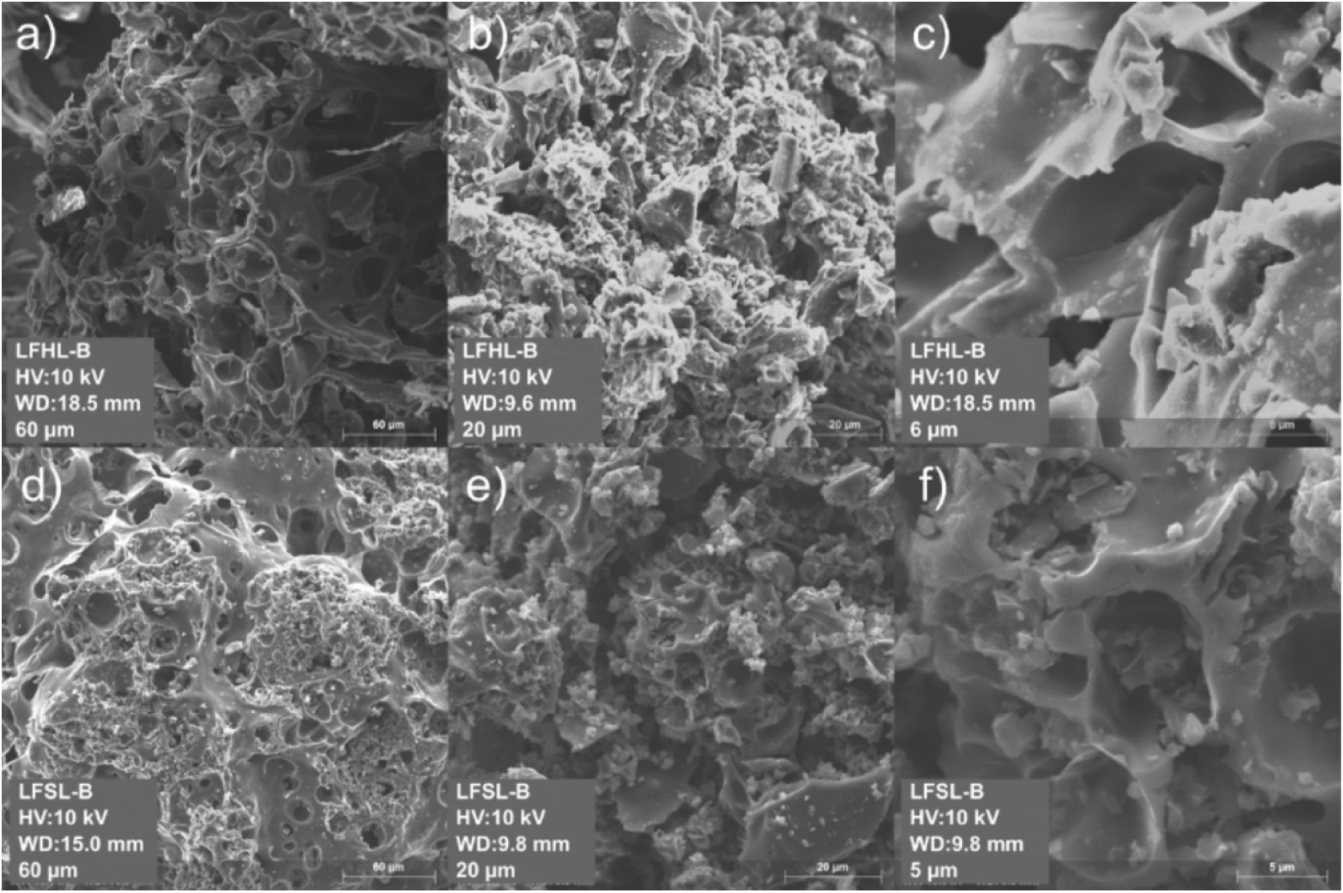
SEM images of LignoForce lignin biochars at different magnifications: (a) LFSL-B, 60 μm; (b) LFSL-B, 20 μm; (c) LFSL-B, 6 μm; (d) LFHL-B, 60 μm; (e) LFHL-B, 20 μm; (f) LFHL-B, 5 μm. Pyrolysis was performed with 30 wt % K_3_PO_4_ at 450 °C under 1000 W microwave power.

MAP biochars seemed to preserve the amorphous nature of the precursor lignins.^39^ LFSL-B and LFHL-B micrographs resembled the alkaline lignin-based biochar produced by conventional pyrolysis at 550 °C for application as high-performance capacitors.^43^ By contrast, Li et al.^44^ produced spherically shaped biochars from a commercially available alkaline lignin (Sigma-Aldrich) in a muffle furnace, using a N_2_ flow of 2 L min^–1^ at 400 °C or 10 °C min^–1^ at 600 °C for 3 h. Therefore, the biochar morphology depends on pyrolysis conditions.

### 3.6 MB Adsorption Dosing Test

LFSL-B and LFHL-B adsorptive capacities were tested with an MB aqueous standard solution. The dosage test demonstrated that 4 g L^–1^ of the adsorbents almost completely removed MB from a 50 mg L^–1^ solution. The remaining dye concentration was determined using a calibration curve ranging from 0.5 to 50 mg L^–1^ (*R* ^2^ = 0.9963). The lowest biochar concentration (0.5 g L^–1^) enabled an MB removal efficiency of 14.6% and 21.8% for LFSL-B and LFHL-B, respectively. Increasing the biochar load to 3.0 g L^–1^ resulted in a removal efficiency higher than 95% in both cases (Figure 6).

**Figure 6:**
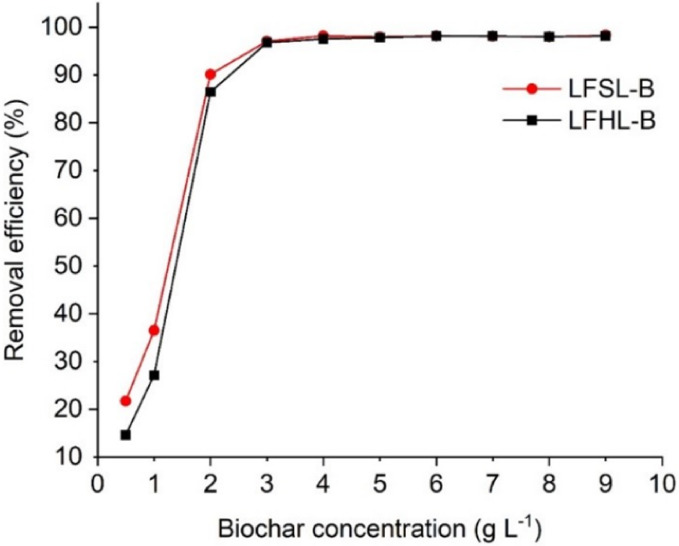
Removal efficiency of biochar using a 50 mL batch of 50 mg L^–1^ MB.

### 3.7 Influence of pH on MB Adsorption

The effect of pH on biochar adsorption performance was investigated at 24 °C using 4 g L^–1^ biochar and a constant MB concentration of 50 mg L^–1^. Changes in pH can affect the biochar surface charge by altering the dissociation of ionizable functional groups. At pH 2, LFSL-B and LFHL-B demonstrated a low MB adsorption capacity of only 5.87 and 8.43 mg g^–1^, respectively (Figure 7a). Low removal efficiencies were observed at this pH (35.9% and 49.8%, respectively). However, when the pH was adjusted to 12, the adsorption capacity of LFHL-B doubled, and that of LFSL-B practically tripled (increased by 2.7 times). At this pH, the adsorption capacity and removal efficiency reached 15.75 mg g^–1^ and 96.3% for LFSL-B and 15.53 mg g^–1^ and 94.5% for LFHL-B, respectively (Figure 7b).

**Figure 7:**
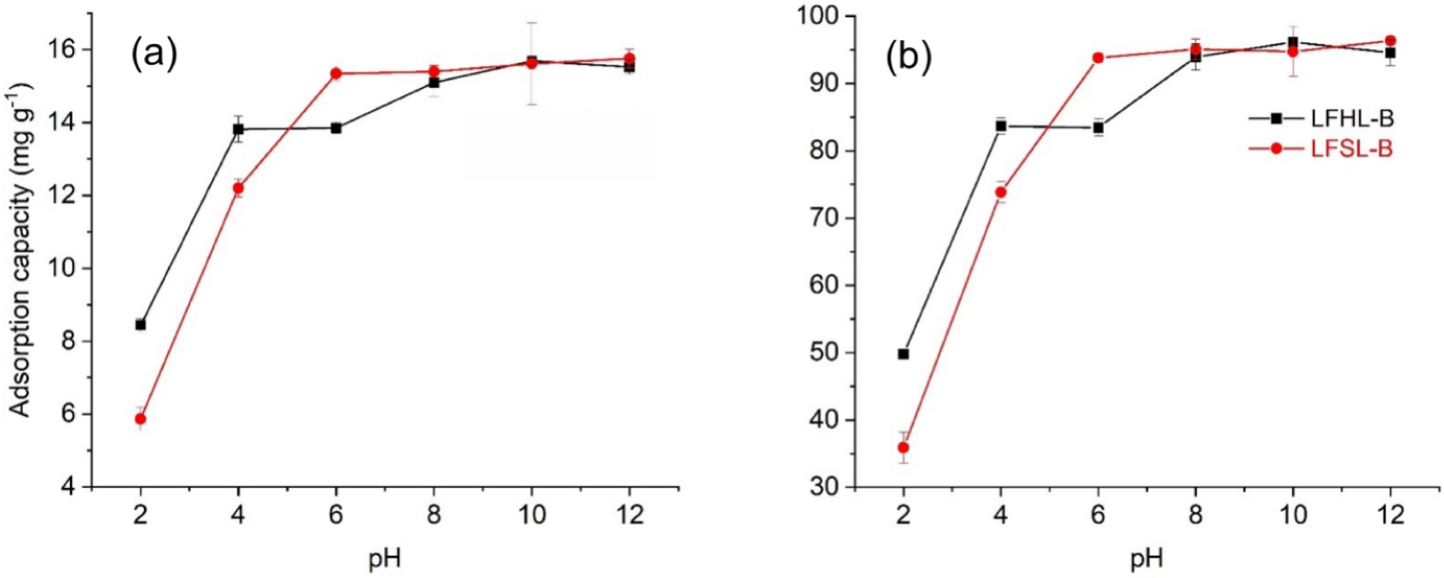
Influence of pH on the MB adsorption capacity (a) and removal efficiency (b) of LignoForce lignin biochars at different pH values, using batch experiments conducted for 60 min at 24 °C and 150 rpm.

The low adsorption capacities of LFSL-B and LFHL-B at low pH values were probably due to repulsive effects among positively charged functional groups. Hence, protonation of functional groups on the adsorbent surface results in the electrostatic repulsion of MB molecules. Carboxylic acids and phenols are fully protonated at pH 2, resulting in inefficient MB adsorption for LFSL-B and LFHL-B. However, for pH values above 4, the adsorbent surface becomes negatively charged, attracting MB molecules and improving the adsorption efficiency to approximately 80%. This significant improvement from pH 3.5 onward indicates the mediation of adsorption by carboxylated substances with p*K*_a_ values around this pH. Therefore, a pH adjustment to values above 7 is critical to ensuring good MB adsorption efficiencies. These results are consistent with previous studies reporting efficient MB adsorption at high pH levels by biochars derived from lignin and chitosan,^50^ bamboo,^51^ and commercial activated carbon.^52^ Alternatively, anionic dyes such as methyl orange are better adsorbed via electrostatic interactions at low pH values.

Under comparable conditions, commercial activated carbons typically exhibit MB adsorption capacities in the range of 200–600 mg g^–1^.^53^ Although the adsorbent produced in our study by lignin pyrolysis falls below this range, it is comparable to the one obtained by many other materials, such as natural zeolites, kaolin, fly ash, or many agricultural solid wastes.^54^

Several studies have reported similar trends. Yu et al.^53^ showed that the adsorption capacity of MB on polymer-modified yeast increased by nearly 4-fold when the pH was raised from 2 to 8. Deng et al.^55^ observed a 2.5-fold increase in MB removal when the pH increased from 2 to 11 for adsorbents derived from cotton stalk, while acid-treated materials showed little sensitivity to pH. Cherifi et al.^56^ reported comparable behavior for vegetal fiber activated carbon, and Wongcharee et al.^57^ found that MB adsorption on a macadamia shell activated carbon–zeolite composite more than doubled when the pH increased from 2 to 8. The underlying mechanism is consistent across these studies. The pH influences both the ionization state of surface functional groups—particularly carboxyl and phenolic groups—and the speciation of cationic dyes.^53^ At low pH, adsorbent surfaces tend to be positively charged, resulting in electrostatic repulsion between the surface and MB, which reduces adsorption. At higher pH, deprotonation of acidic surface groups increases the negative surface charge, thereby enhancing electrostatic attraction and promoting MB uptake. Xia et al.^58^ further emphasized that higher pH conditions stabilize coordination interactions between MB and the adsorbent’s functional groups, contributing to improved adsorption performance.

### 3.8 Adsorption Kinetics and Equilibrium Time

The adsorption kinetics were investigated to evaluate the rate of uptake and establish the time required to reach equilibrium. For the LFSL-B sample, the kinetic profile showed a rapid initial adsorption, followed by a slower phase as it approached equilibrium. Although at 60 min the system had not fully reached the steady-state plateau, it was sufficiently close to equilibrium conditions to allow for representative isotherm analysis. In contrast, the LFHL-B sample exhibited more complex kinetic behavior. Traditional pseudo-first- and pseudo-second-order models failed to provide an adequate fit for this material, requiring a higher-order (7th) equation to accurately describe the experimental data and reduce error.

The kinetic data are represented using Figure 8 to better visualize the trend without imposing a biased physical model. On the basis of these kinetic results, it was assumed that most (95% for LFSL-B, 99% for LFHL-B) of the adsorption capacity was captured for both materials while maintaining the same experimental conditions.

**Figure 8:**
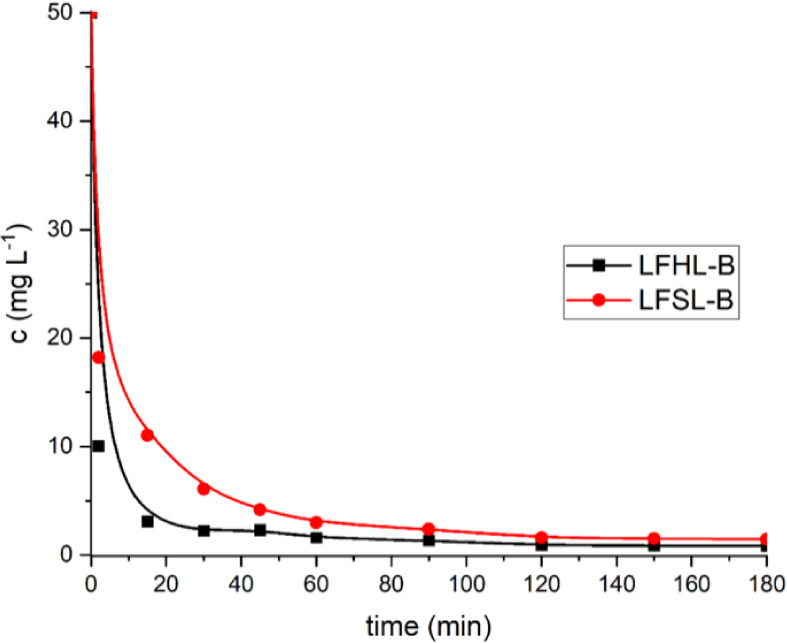
Kinetic information about MB adsorption by kraft lignin biochars.

### 3.9 Adsorption Isotherms

Although kinetic data indicated that full equilibrium was not reached at 60 min for all samples, the isotherm models (Langmuir, Freundlich, and Jovanović) were fitted to the experimental data to determine the apparent adsorption capacity. This provides a reliable comparison of the materials’ performance under consistent operational conditions (Figure 9). Both lignin biochars had similar adsorption isotherms, with the Jovanović model providing the best fit, as seen by the lowest Mean Square Errors (MSE) in Table 3. Therefore, this model was deemed best for describing the MB adsorption behavior under operational conditions.

**Figure 9:**
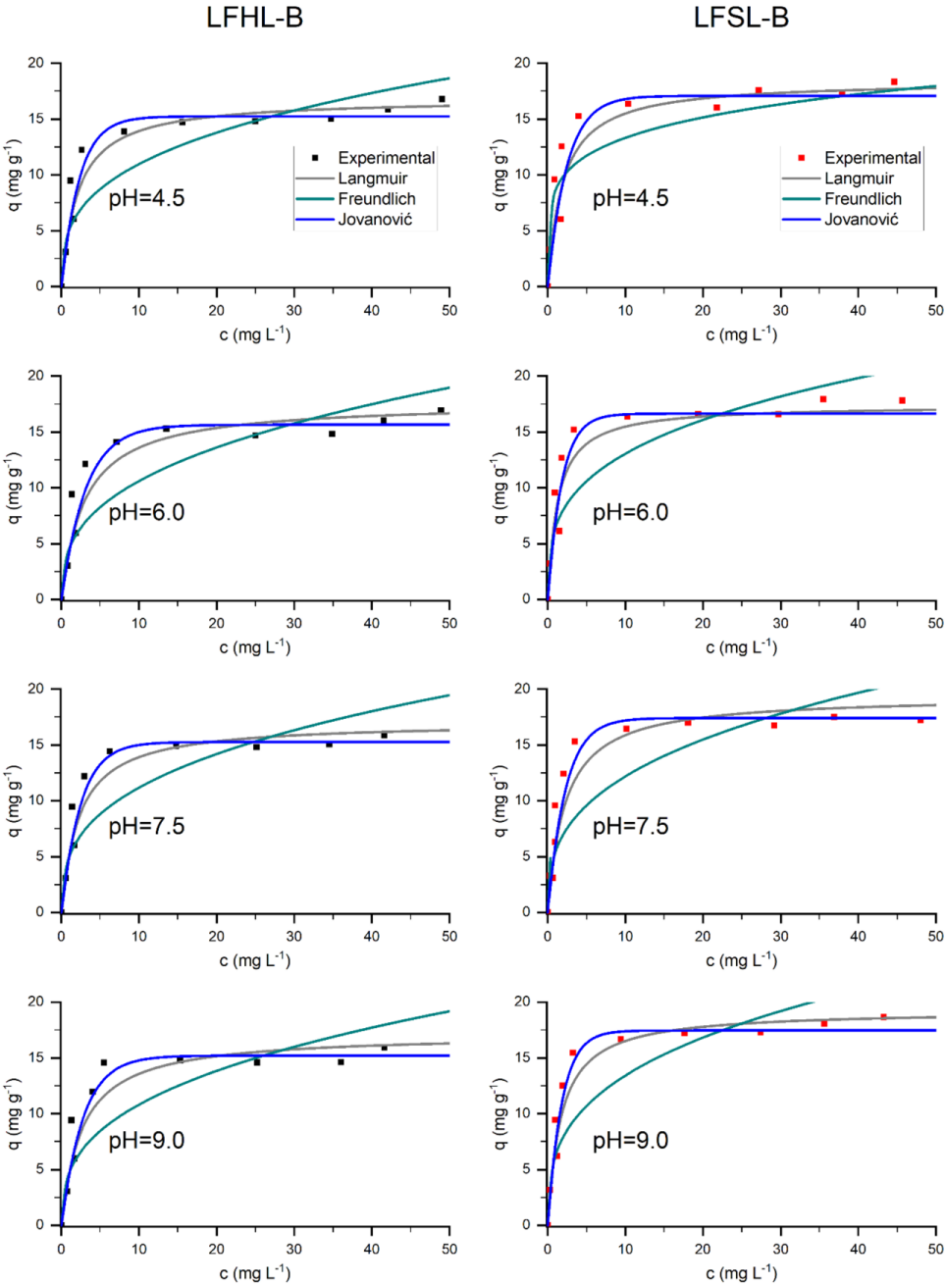
Experimental and fitted isotherms for the adsorption of methylene blue on LignoForce lignin biochars at pH values of 4.5, 6.0, 7.5, and 9.0.

**Table 3:** Parameters of the Langmuir, Freundlich, and Jovanović Models for MB Adsorption by LignoForce Lignin Biochars at Different pH Values

	Langmuir			Freundlich			Jovanović		
pH	Parameter	LFSL-B	LFHL-B	Parameter	LFSL-B	LFHL-B	Parameter	LFSL-B	LFHL-B
4.5	q_m_ (mg g^–1^)	18.52	16.51	K_F_ (mg g^–1^)	8.69	5.12	q_m_ (mg g^–1^)	17.1	15.3
	K_L_ (L mg^–1^)	0.52	0.57	1/n	0.186	0.331	K_J_ (L mg^–1^)	0.407	0.433
	MSE	0.182	0.0316	MSE	0.0679	0.0768	MSE	0.185	0.0296
6.0	q_m_ (mg g^–1^)	18.32	16.93	K_F_ (mg g^–1^)	6.53	4.62	q_m_ (mg g^–1^)	16.7	15.7
	K_L_ (L mg^–1^)	0.59	0.45	1/n	0.301	0.361	K_J_ (L mg^–1^)	0.558	0.318
	MSE	0.0601	0.0418	MSE	0.0735	0.0927	MSE	0.0772	0.0347
7.5	q_m_ (mg g^–1^)	18.29	16.80	K_F_ (mg g^–1^)	5.50	5.05	q_m_ (mg g^–1^)	17.4	15.3
	K_L_ (L mg^–1^)	0.77	0.51	1/n	0.346	0.345	K_J_ (L mg^–1^)	0.439	0.405
	MSE	0.0521	0.0322	MSE	0.123	0.0884	MSE	0.0375	0.0262
9.0	q_m_ (mg g^–1^)	19.27	16.37	K_F_ (mg g^–1^)	6.34	4.76	q_m_ (mg g^–1^)	17.5	15.2
	K_L_ (L mg^–1^)	0.655	0.51	1/n	0.326	0.356	K_J_ (L mg^–1^)	0.551	0.357
	MSE	0.0316	0.0402	MSE	0.0777	0.0980	MSE	0.0343	0.0309

The partial fit of the Langmuir isotherm indicates that the adsorption sites are evenly distributed across the biochar surface, resulting in a monolayer adsorption pattern. By contrast, the Freundlich model could not adequately fit the experimental data, but 1/n values lower than unity suggested a favorable adsorption process. Although less commonly used than the Langmuir isotherm, the Jovanović model is also based on the type I monolayer adsorption theory but additionally accounts for collisions between adsorbing and desorbing molecules, allowing readsorption (a feature not included in the standard Langmuir model).^59^ This leads to earlier saturation at lower solute concentrations and can better capture dynamic adsorption equilibria on surfaces with variable potential. Wongcharee et al.^57^ observed the same trend for MB adsorption on a mesoporous activated carbon–zeolite composite. The model allows hopping of adsorbed molecules to vacant neighboring sites while excluding lateral interactions, reflecting the heterogeneity of the surface and the distribution of adsorption energies. While the Jovanović isotherm may not always yield more accurate predictions for MB adsorption, poor fits are often reported due to the improper use of the model.^59^

Unlike the Langmuir model, the Jovanović model accounts for surface heterogeneity. This model assumes that the periodicity of the crystal lattice or the porous surface creates preferred sites for adsorption, even on a homotactic (isoenergetic) surface where the energy of the adsorbent molecules is at its lowest state.^60^ Notably, the adsorption values predicted by this model agreed with the experimentally determined values at pH 6.0 and 7.5. However, minor differences were observed at pH 4.5 and 9.0, indicating that the Langmuir model could also describe the adsorption phenomenon under these conditions.

MB adsorption by LignoForce lignin biochars can be attributed to various mechanisms, including electrostatic interactions, hydrogen bonding, and π-π interactions with the cationic dye.^28^ Furthermore, the presence of mesopores in LFSL-B and LFHL-B enhances the adsorption capacity of MB because it fits into both microporous and mesoporous cavities.^61,62^ The biochar adsorption capacity can be further improved through activation strategies. Wang et al.^51^ demonstrated that using K_2_CO_3_ to activate bamboo waste-derived biochar resulted in a specific surface area of 1565 m^2^ g^–1^ with an MB adsorption capacity of 1100 mg g^–1^. Shi et al.^29^ improved the adsorption efficiency of waste palm shell biochar using physicochemical activation, which produced a surface area of 717.8 m^2^ g^–1^ and a dechlorination efficiency of 35.8 mg g^–1^. Activation also played an outstanding role in the MB adsorption properties of potato peel biochar, which reached adsorption capacities of 1246 mg g^–1^. In this way, LignoForce lignin biochars could be improved to broaden their application scope.

## 4 Conclusion

The catalytic microwave-assisted pyrolysis (MAP) of hardwood and softwood LignoForce lignins was performed. K_3_PO_4_ functioned as both a microwave absorber and a catalyst to enhance biochar microporosity. A detailed comparative analysis of the thermal behavior of lignin biochars (LFHL and LFSL) under microwave irradiation revealed a correlation between distinct heating rates and their specific chemical compositions (e.g., presence of β-O-4’ and β- β’ linkages). Even at a moderate 450 °C, the catalytic MAP yielded biochars with substantial specific surface areas (142 and 202 m^2^ g^–1^, respectively). Both kraft lignin biochars (LFHL-B and LFSL-B) displayed amorphous structures with suitable surface properties for the adsorption of cationic dyes such as methylene blue (MB). pH influenced the biochar adsorption efficiency, emphasizing electrostatic attraction as the primary interaction. Adsorption isotherms adhered well to the Jovanović model, accommodating pore heterogeneity. The kinetic data revealed that LFHL-B outperformed LFSL-B for MB adsorption, but both were able to remove over 95% of MB from an aqueous solution, particularly at pH values higher than 4.5. These results position kraft lignin biochars as valuable carbon-accumulating raw materials for environmental applications and establish K_3_PO_4_-catalyzed MAP as a fast and energy-efficient route for their thermal conversion.

## Supplementary Material



## Notes

Figure S1 – N_2_ adsorption and desorption isotherms for LFHL-Biochar and LFSL-Biochar. Figure S2 – Pore size distribution by the BJH method for LFSL-B and LFHL-B over a micromesoporous range (PDF)
