# Supplementary material for: Carbonaceous Materials for Wastewater Treatment from Microwave-Assisted Pyrolysis of LignoForce Lignins
Source: ACS Omega. 2026 Jun 8;11(24):35047–57. doi: 10.1021/acsomega.5c11603 (PMC13294895; doi:10.1021/acsomega.5c11603)
Supplement: Supplementary file 1 [file ao5c11603_si_001.pdf]

SUPPORTING INFORMATION

for

CARBONACEOUS MATERIALS FOR WASTEWATER TREATMENT FROM MICROWAVE-ASSISTED PYROLYSIS OF LIGNOFORCE™ LIGNINS

Maria J. Suota [†*], *Mohammad S. Ghazani*[‡], *Gorka Elordi*[§], *Jie Wu*[þ], *Jack Saddler* [þ], *Xiaotao Bi*[‡], *Luiz P. Ramos* [†1] [†]Graduate Program in Chemistry, Federal University of Paraná, Curitiba, 81531-980, Brazil

[‡]Chemical and Biological Engineering Department, University of British Columbia, Vancouver, BC V6T 1Z3, Canada

[§]Chemical Engineering Department, University of the Basque Country, Basque Country, Spain

[þ]Faculty of Forestry, University of British Columbia, Vancouver, Canada Department of Wood Science, Faculty of Forestry, The University of British Columbia, 2424 Main Mall, Vancouver, BC, Canada

SUPPORTING INFORMATION

Figure S1 – N_2 adsorption and desorption isotherms for LFHL-Biochar and LFSL-Biochar.

Figure S2 – Pore size distribution by the BJH method for LFSL-B and LFHL-B over a micro-mesoporous range.

[1] Corresponding authors: luiz.ramos@ufpr.br

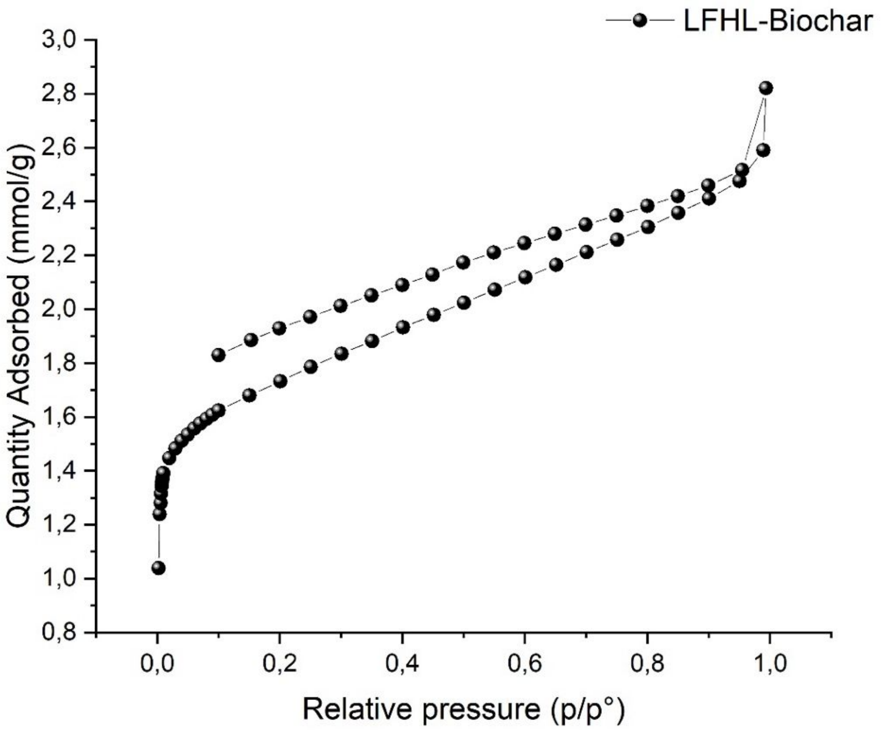

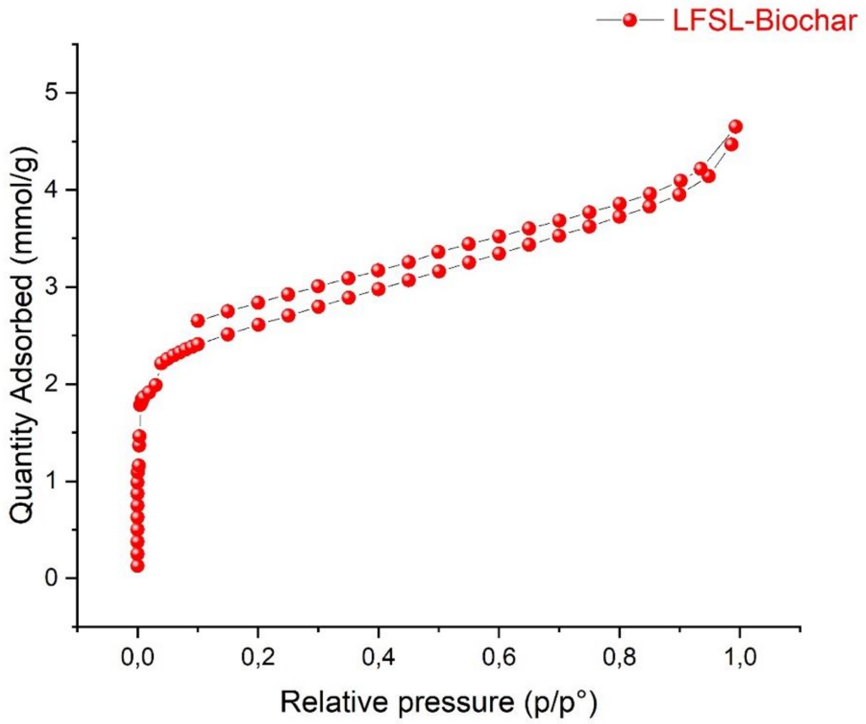

Figure S1

N_2 adsorption and desorption isotherms for LFHL-Biochar and LFSL-Biochar.

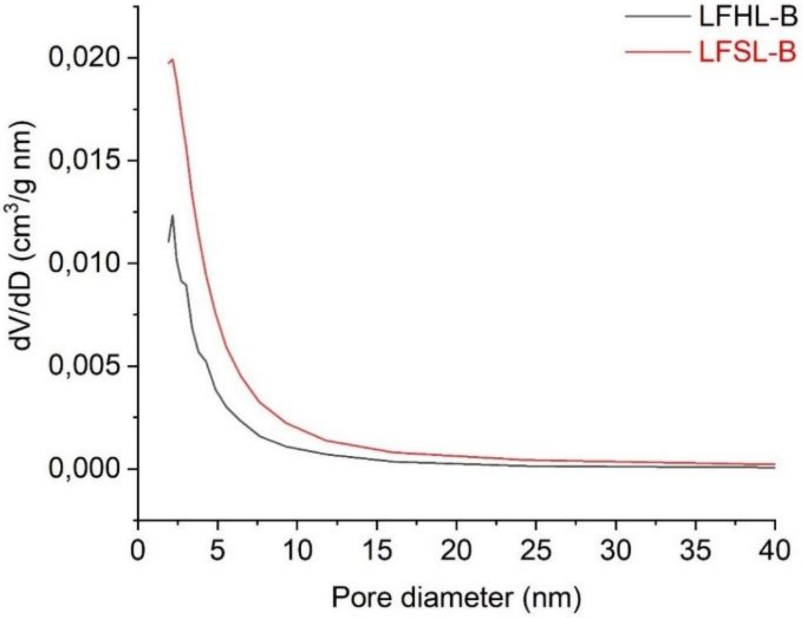

Figure S2

Pore size distribution by the BJH method for LFSL-B and LFHL-B over a micro-mesoporous range.
